# Fibromyalgia is associated with hypersensitivity but not with abnormal pain modulation: evidence from QST trials and spinal fMRI

**DOI:** 10.3389/fpain.2023.1284103

**Published:** 2023-12-05

**Authors:** Roland Staud, Melyssa M. Godfrey, Patrick W. Stroman

**Affiliations:** ^1^ Division of Rheumatology and Clinical Immunology, University of Florida, Gainesville, FL, United States; ^2^ Center for Neuroscience Studies, Queen’s University, Kingston, ON, Canada

**Keywords:** chronic pain, fibromyalgia, central sensitization, QST, conditioned pain modulation

## Abstract

Widespread pain and hyperalgesia are characteristics of chronic musculoskeletal pain conditions, including fibromyalgia syndrome (FM). Despite mixed evidence, there is increasing consensus that these characteristics depend on abnormal pain augmentation and dysfunctional pain inhibition. Our recent investigations of pain modulation with individually adjusted nociceptive stimuli have confirmed the mechanical and thermal hyperalgesia of FM patients but failed to detect abnormalities of pain summation or descending pain inhibition. Furthermore, our functional magnetic resonance imaging evaluations of spinal and brainstem pain processing during application of sensitivity-adjusted heat stimuli demonstrated similar temporal patterns of spinal cord activation in FM and HC participants. However, detailed modeling of brainstem activation showed that BOLD activity during “pain summation” was increased in FM subjects, suggesting differences in brain stem modulation of nociceptive stimuli compared to HC. Whereas these differences in brain stem activation are likely related to the hypersensitivity of FM patients, the overall central pain modulation of FM showed no significant abnormalities. These findings suggest that FM patients are hyperalgesic but modulate nociceptive input as effectively as HC.

## Introduction

Pain was defined by the International Association for the Study of Pain (IASP) as an unpleasant sensory and emotional experience associated with, or resembling that associated with, actual or potential tissue damage (1). Nociceptive pain depends on activation of nociceptors in peripheral tissues to direct individuals’ attention on impending injury and to elicit adaptive behaviors. Pain can be divided into acute pain, which is usually due to a recent or pending injury, and chronic pain which lasts for more than 3 months (2). Pain was labelled as chronic primary pain by the International Classification of Diseases (ICD-11) (3) when pain persists for more than 3 months and is associated with significant emotional distress and/or functional disability, and the pain is not better accounted for by another condition. Examples of chronic primary pain include chronic primary headache, complex regional pain syndrome (CRPS), chronic orofacial pain, chronic primary visceral pain, chronic widespread pain, and fibromyalgia (FM). These chronic pain conditions also fulfill the nociplastic pain classification criteria proposed by the IASP (4).

### Nociplastic pain

The peripheral and central nervous system constantly adapts to intrinsic and extrinsic challenges. This neuronal plasticity can result in increased or reduced responsiveness of neurons to peripheral nociceptive input. Strong or prolonged nociceptive input can lead to peripheral and/or central sensitization which is characterized by increased responding of nociceptive neurons in the peripheral and central nervous system (CNS), respectively (4).

In 2021, the International Association for the Study of Pain created the new term “nociplastic pain” for chronic musculoskeletal pain that appears to be out of proportion to detectable tissue injuries. Patients with nociplastic pain often report insomnia, excessive fatigue, and cognitive abnormalities, as well as hypersensitivity to sound, light, taste, and odor (5). Some of the most prevalent nociplastic pain conditions include tension-type headache, chronic pelvic pain, chronic low back pain and FM. Although the exact mechanisms of nociplastic pain is unclear many investigators consider central pain processing abnormalities including abnormal pain modulation, as relevant (6). Furthermore, numerous findings of brain abnormalities have been associated with nociplastic pain, including changes in the grey matter volume and altered functional connectivity of brain regions involved in pain and sensory processing (7). Increasing evidence also suggests that neuroinflammation, characterized by glial activation, production of neurotransmitters, and proinflammatory cytokines, may also play an important role for nociplastic pain (8–10).

Although the term “nociplastic” pain was meant to describe complex chronic pain conditions like FM, irritable bowel syndrome, temporomandibular disorder, and chronic back pain (11), it is controversial. Neuroplasticity is not a novel concept as both peripheral and central sensitization depend on neuroplasticity associated with multiple translational and transcriptional changes of neurons in the peripheral and central nervous system (CNS) (4). Whereas the evidence for peripheral and central sensitization is well established in inflammatory or neuropathic pain conditions the mechanisms of nociplastic pain are not as well understood. We have recently shown that the pain of FM patients (or nociplastic pain) is due to hypersensitivity of central nervous system pathways and not to augmented pain processing in the dorsal horn of the spinal cord or brain as previously thought (12, 13).

### Pain modulation during acute or chronic musculoskeletal pain

#### Peripheral sensitization

Although not considered a major contributor to chronic pain, peripheral sensitization can frequently be detected in patients with chronic musculoskeletal pain syndromes (14, 15) After repeated or intense noxious stimuli, sensitization of peripheral afferents can occur, resulting in decreased activation thresholds and amplified responses to subsequent inputs (16–18). Without continuing nociceptive input, increased pain sensitivity usually normalizes over time, making this phenomenon sometimes long lasting but reversible. Besides increased excitability of primary sensory neurons, increased expression of ion channels on nerve endings and axons can occur, including potassium channels (19), voltage-gated sodium (Nav) channels (20), voltage-gated calcium (Cav) channels (21), acid-sensing ion channels (ASICs) (22). and transient receptor potential (TRP) channels (23, 24).

#### Central sensitization

Central sensitization occurs mostly at the dorsal horn neuron level and is usually associated with acute tissue injury. It is a complex neurophysiological process that has been thought to be responsible for the increased pain sensitivity of many patients with chronic pain syndromes, including FM. It involves changes in the function and connectivity of dorsal horn neurons of the spinal cord, which play a critical role in processing and transmitting painful signals to the brain (25). Central sensitization involves an increase in the excitability of nociceptive neurons and this heightened excitability can be attributed to various mechanisms, including changes in the ion channels, particularly after activation of N-Methyl-D-Aspartate (NMDA) receptors. Central sensitization can also lead to the expansion of the receptive fields of dorsal horn neurons. Normally, these neurons respond to a specific area of the body, but in central sensitization, their responses can spread to adjacent or even distant regions. This can result in pain spreading beyond the initial injury site.

The lack of consistent evidence for a major role of peripheral sensitization in chronic musculoskeletal pain syndromes has focused increased attention on central pain amplification. Central sensitization has provided a mechanistic explanation for some of the temporal, spatial, and sensitivity changes in acute and chronic pain syndromes like FM and has focused attention on the important contribution of the central nervous system to the increased pain sensitivity of chronic pain patients. Whereas peripheral sensitization is characterized by reduced pain thresholds and hypersensitivity of local nociceptors (26–28), central sensitization causes pain hypersensitivity in areas outside of tissue injury and requires little peripheral input to maintain high pain sensitivity even after tissue healing has occurred. Although central sensitization clearly plays an important role for pain reported after tissue injury, its particular role in many patients with “nociplastic” pain syndromes, like FM is unclear.

Animal experiments have identified two major forms of pain sensitivity augmentation: central sensitization (CS) which occurs primarily in the spinal cord and long-term potentiation (LTP) which affects preferentially brain regions. CS is frequently present after tissue injury and LTP is a critical process for memory formation but also for chronic pain. CS comprises augmented responses of dorsal horn neurons after electrical nerve stimulation associated with long-lasting aftersensations (14). Its neural correlate in human subjects is “windup” which is associated with altered functional connectivity of regions involved in pain and sensory processing (7, 29). Spinal modulation of nociceptive input occurs in the dorsal horn of the spinal cord where incoming signals are transmitted to interneurons in Rexed laminae before they are projected to the brain. Central sensitivity changes can involve excitatory neurotransmitter release comprising glutamate and aspartate, or inhibitory neurotransmitters including GABA and glycine (30).

### Long-term potentiation

The central nervous system is a complex network and its functioning depends on the excitability of individual neurons but the strength of their synaptic connections can vary (31). Such neuronal plasticity is crucial for effective nervous system functioning including memory formation (32) and chronic pain (33). Some forms of neuronal plasticity are due to long-term potentiation (LTP) which plays not only an important role in memory consolidation (34), but also for pain (35). Usually LTP of spinal neurons occurs during high frequency electrical stimulation (33, 36), but can also be induced by tissue inflammation (37), and peripheral nerve injury (38).

### Descending pain modulation

The dorsal horn of the spinal cord comprises 2nd order neurons which project to the brainstem and brain. Descending modulation from the midbrain periaqueductal gray and the rostral ventromedial medulla (RVM) affects the activity of these neurons as either pain inhibitory or facilitatory (39) through release of multiple neurotransmitters including serotonin, noradrenaline, and dopamine (40). Abnormalities of descending pain modulation, including pain facilitation and pain inhibition can be evaluated by quantitative sensory tests like temporal summation of pain (TSP) (41, 42) and conditioned pain modulation (CPM), respectively (43).

It is well known that cognitions and feelings can influence pain perception (44). Previous research has demonstrated that distractions can reduce pain intensity, whereas attention to a nociceptive stimulus will increase the pain perceived (45). Furthermore, interventions that elicit positive affect may result in reduced pain perception, whereas the presence of negative affect may increase pain intensity (46). Previous findings seemed to indicate that descending pain modulation is dysfunctional in chronic pain conditions such as FM, temporomandibular disorder, irritable bowel syndrome and chronic headaches (47). Therefore, dysfunctional pain modulation seemed to contribute to the development and maintenance of chronic pain syndromes (48). Descending pain control from the brain stem affects the entire body (49) and can dramatically increase the pain sensitivity of individuals with chronic pain (50, 51). However, not all studies of chronic pain patients reported abnormal descending pain modulation. Normal pain modulation was reported in some studies of patients with chronic low back pain (52, 53).

### Neuro-inflammation

Glial cells are critical for structure and function of the brain (54). Proinflammatory cytokine signaling between glial cells provides protection from infection and plays an important role in tissue repair and recovery (55). Glia can be activated by many cytokines, chemokines, proteases, and growth factors, that not only play an important role for chronic pain (56) but are also important for memory (57) and pain modulation (58). For example, cytokine inhibition has been found to improve osteoarthritis pain (59), low back pain (60), and rheumatoid arthritis pain (61). Therefore, excessive glial activation (neuroinflammation) may play an important role in chronic pain states and decreasing neuroinflammation may be a promising treatment target for patients with such conditions (62). Previous positron emission tomography (PET) studies of chronic pain patients reported widespread cortical enhancements consistent with neuroinflammation. For example, when the [11C]PBR28 PET ligand which binds to the translocator protein (TSPO) on activated microglia or astrocytes (63), was used in FM patients neuroinflammation could be observed in M1, S1, precuneus, superior parietal lobe, insula, and thalamus (64, 65). In another study of FM patients, increased TSPO binding could be demonstrated in the medial and lateral areas of the frontal and parietal lobe (66) and fatigue ratings of FM patients correlated with [11C]PBR28 binding in the anterior and posterior middle cingulate cortices.

### Nociplastic pain in FM and other chronic musculoskeletal pain syndromes

Pain is a multifactorial experience associated with memory as well as psycho-social factors (67, 68). While acute pain will focus the individual on the noxious stimuli thus preventing further tissue damage (69), chronic pain is considered “maladaptive” and almost always associated with neuronal plasticity. In chronic musculoskeletal pain conditions, like FM or low back pain, painful sensations are considered nociplastic and strongly associated with hyperalgesia/allodynia (11).

Other views of chronic (nociplastic) pain are centered on stress evoked, sympathetically maintained, neuropathic pain which depends on dorsal root ganglia abnormalities as critical for its pathogenesis (70). This hypothesis is based on evidence that nociplastic pain has some neuropathic features, including paresthesias and allodynia. In some studies, nearly half of FM patients demonstrated evidence of peripheral nerve damage including small nerve fiber pathology (71). However, this hypothesis fails to explain the chronic pain mechanisms in the majority of FM patients.

### Pain facilitation and inhibition are not abnormal in FM patients

FM is a complex syndrome that is comprised of widespread pain, fatigue, insomnia and cognitive difficulties. Its pathogenesis as a central nervous system (CNS) disorder is widely accepted and patients with this syndrome demonstrate hypersensitivity to painful and non-painful stimuli. The symptoms of FM are unspecific and can be found in many other chronic pain disorders including migraine, temporo-mandibular disorder (TMD) and chronic low back pain (72). Enhanced pain facilitation, together with other factors like negative affect, may also help explain the known discordance between the severity of tissue abnormalities and clinical pain associated with FM, OA, and low back pain (73, 74). Deficient descending pain inhibition (Conditioned Pain Modulation: CPM) has been reported in FM (75) but also in other chronic pain conditions, like IBS (76), migraine (47), and low back pain (77). However, high variability of the CPM efficacy of chronic pain patients was noted in previous studies which may be related to the experimental conditions used, including the application of non-standardized conditioning (78) and test (79) stimuli.

Although dysfunctional pain modulation has been proposed as a relevant pathogenetic mechanism of FM, this view has recently become controversial. Therefore, we examined pain modulation of FM patients in several studies, using only experimental stimuli that were adjusted to each participant's pain sensitivity thus eliminating ceiling and floor effects associated with non-standardized stimuli (12, 13, 80).

**Figure 1:**
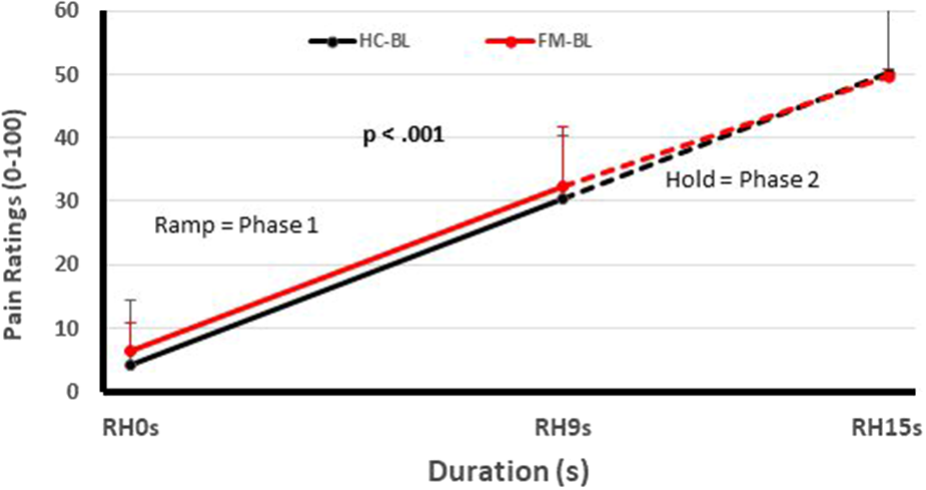
Testing of pain facilitation of HC and FM participants. All subjects received sensitivity-adjusted Ramp&Hold (RH) stimuli at the hand. Pain ratings of the second phase of RH (dotted lines) depend on each individual's pain summation ability. Although pain ratings of FM participant and HC increase significantly during RH (*p* < 0.001), their rate of temporal summation in phase 2 was not significantly different from each other (*p* > 0.05). (RH, ramp & hold; s, seconds; HC, healthy controls; FM, fibromyalgia) [Reproduced with permission (12)].

**Figure 2:**
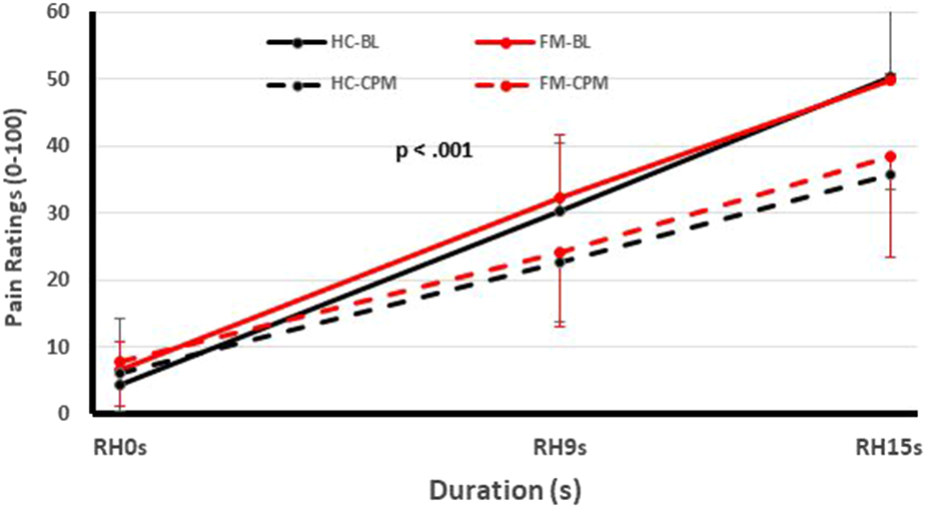
Testing of pain inhibition of FM participants and HC using heat Ramp&Hold (RH) applications to one hand (test stimuli) during immersion of the other hand in a cold water bath (conditioning stimulus). Time course of Ramp&Hold (RH) ratings of FM and HC during baseline (solid lines) and CPM (dotted lines). During baseline (solid lines), the RH ratings of both groups increased significantly (*p* < 0.001) but this increase was not different for HC and FM participants (*p* > 0.05). During CPM (dotted lines), the RH ratings increased significantly less than during baseline testing in HC and FM subjects (*p* < 0.03), but there was no significant difference of CPM efficiency between groups noted (*p* > 0.05). (FM, fibromyalgia; HC, healthy control; CPM, conditioned pain modulation; RH, ramp & hold; BL, baseline; s, second) [Reproduced with permission (12)].

One of these studies (participants *n* = 51) (12) examined the pain inhibitory and facilitatory capacity of FM patients (Figures 1, 2), using such standardized pain stimuli (12). This study demonstrated effective pain inhibition in 87% of FM patients similar to HC. Only 13% of participants failed to show pain reductions or demonstrated pain facilitation. During testing of pain facilitation, FM patients showed normal TSP similar to HC (*p* > 0.05). These results differ from the findings of some other FM studies of pain modulation (75, 81). One reason for the difference of our results in regards to several previous FM studies (52, 82) may be that we applied only test stimuli that were carefully adjusted to each participant's pain sensitivity, thus avoiding ceiling and floor effects. These results provided strong evidence that FM patients can effectively modulate experimental pain similar to HC.

### Pain hypersensitivity is a key feature of FM

Over the last decade, our understanding of chronic pain has significantly advanced thanks to behavioral and brain imaging studies (83). Some trials suggested that FM is associated with abnormal resting-state functional connectivity (84) and decreased gray matter of pain processing brain regions (85). Our understanding of pain advanced significantly with the discovery of several brainstem regions that can control pain-related activity in the spinal cord (SC) (50, 83). Several brain and brain stem regions can modulate the activity of dorsal horn neurons through descending modulation, resulting in either pain inhibition or facilitation (86). These areas comprise the periaqueductal gray matter (PAG) and the rostral ventromedial medulla (RVM), which connect with dorsal horn neurons of the SC (87). Because our understanding of pain modulatory processes, particularly in the brainstem and spinal cord, was mostly derived from animal studies (88, 89), we examined the pain modulation of chronic pain patients using functional magnetic resonance imaging of the spinal cord. Our studies (participants *n* = 59) showed that FM patients not only failed to activate some regions of the descending pain modulatory system but also demonstrated lower connectivity to other pain modulatory areas including the amygdala, hippocampus, and brainstem (80, 90). However, when we tested spinal cord activity of FM patients during the application of standardized experimental pain stimuli, the spinal cord BOLD responses of FM patients were similar to those of HC, indicating that pain related spinal cord activity of FM patients was similar to HC (83, 91). Statistical modeling of pain related CNS activation demonstrated different connectivity of FM patients to/from the PAG compared to HC and was associated with participants’ pain ratings. Thus, despite demonstrating similar efficacy of pain facilitation and inhibition compared to HC, FM participants showed some differences of descending pain regulation via the PAG-RVM-spinal cord pathway. At least some of these differences may be associated with the hyperalgesia of FM participants.

## Caveats

Experimental pain studies play a crucial role in advancing our understanding of pain modulation and its relevance to chronic pain. While these studies provide valuable insights, researchers must continue to bridge the gap between experimental findings and the complex pain experiences of chronic pain patients to develop more effective and tailored pain management strategies. Interdisciplinary approaches that combine neurobiology, psychology, and clinical practice are key to this endeavor.

## Conclusions

The presumption that abnormal pain facilitation and inhibition are major contributors to FM pathogenesis remains controversial. No significant differences in pain facilitation or inhibition could be detected between FM patients and HC in one of our recent investigations when quantitative sensory testing was performed using standardized nociceptive stimuli (12). Given that the detection of effective pain modulation by CPM seems to be dependent on stimulus modality, heat vs. pressure (79), our investigation using predominantly heat stimuli may have facilitated our ability to detect normal pain modulation of FM patients. Our conclusions are also supported by several brainstem and spinal cord imaging investigations of FM patients and HC which demonstrated similar spinal cord activity in both groups during pain facilitation with standardized heat pain stimuli (13, 80). This lack of spinal cord functional abnormalities in FM seems to emphasize brain and brainstem functional alterations as more important for the pain and hyperalgesia of these patients and not abnormal pain modulation. There is increasing evidence for such functional brain abnormalities in FM (92–95) but the specificity of these findings is still unclear. Overall, the results of our studies seem to suggest that not only pain facilitation but also pain inhibition are normal in FM patients. Our findings also indicate that one of the hallmarks of FM and possibly of other chronic musculoskeletal pain syndrome is hyperalgesia and not abnormal pain modulation. Future studies need to focus on CNS mechanisms that are responsible for FM patients’ hypersensitivity to sensory stimuli which is not limited to nociceptive input but extends to most other sensory domains including sound, light, and sense of smell.
